# Chromosome distribution in human sperm – a 3D multicolor banding-study

**DOI:** 10.1186/1755-8166-1-25

**Published:** 2008-11-14

**Authors:** Marina Manvelyan, Friederike Hunstig, Samarth Bhatt, Kristin Mrasek, Franck Pellestor, Anja Weise, Isabella Simonyan, Rouben Aroutiounian, Thomas Liehr

**Affiliations:** 1 Department of Genetic and Laboratory of Cytogenetics, State University, Yerevan, Armenia; 2 Research Centre of Maternal and Child Health Protection, Yerevan, Armenia; 3 Institute of Human Genetics and Anthropology, Jena, Germany; 4 INSERM U847, Montpellier, France; 5 University of Montpellier I, Montpellier, France; 6 Baylor College of Medicine, Houston, Texas, USA; 7 Department of Reproduction biology, CHU Montpellier, Montpellier, France

## Abstract

**Background:**

Nuclear architecture studies in human sperm are sparse. By now performed ones were practically all done on flattened nuclei. Thus, studies close at the *in vivo *state of sperm, i.e. on three-dimensionally conserved interphase cells, are lacking by now. Only the position of 14 chromosomes in human sperm was studied.

**Results:**

Here for the first time a combination of multicolor banding (MCB) and three-dimensional analysis of interphase cells was used to characterize the position and orientation of all human chromosomes in sperm cells of a healthy donor. The interphase nuclei of human sperm are organized in a non-random way, driven by the gene density and chromosome size.

**Conclusion:**

Here we present the first comprehensive results on the nuclear architecture of normal human sperm. Future studies in this tissue type, e.g. also in male patients with unexplained fertility problems, may characterize yet unknown mechanisms of infertility.

## Background

Interphase chromosome organization and nuclear architecture are already being investigated for a long time [1-3]. Chromosomes have been demonstrated to be located in specific regions in the interphase nucleus. These were called 'chromosome territories' [4-7]. However, our own multicolor banding (MCB) based studies [8] showed, that the chromosome shape is not lost in the interphase nucleus and one can even identify interphase chromosomes instead of only chromosome territory [9-11]. MCB is the only approach available at present that provides the possibility of characterizing the chromosomal integrity of arbitrary interphase cell populations [12,13]. It is still a matter of discussion what influences more the nuclear position of chromosomes: chromosome size or gene density. It has been repeatedly shown that small chromosomes preferentially locate close to the center of the nucleus, while large chromosomes can be found in the nuclear periphery of human fibroblasts [11-15]. Nonetheless, also evidence for a gene density-correlated radial arrangement of chromosomes in the nucleus was provided [16]. Human chromosome #19, which is gene-dense and early replicating shows a localization in the central part, for the approximately same sized chromosome 18 a localization in the peripheral part of the nucleus was repeatedly proven. As the latter is gene-poorer and comprises late-replicating chromatin this gene-density factor is often discussed as a general principle, also as this nuclear topological arrangement was conserved during evolution [11,6-19].

Three-dimensional (3D) FISH analysis became a major tool for studying the high order chromatin organization in the cell nucleus [20,21]. However, up to now only one 3D-study is available for sperm [22]. In the present study the MCB-based [5] analysis on 3D preserved sperm was performed using suspension fluorescence *in situ *hybridization (S-FISH) [11,23].

## Results and discussion

### MCB studies combined with S-FISH

**Figure 1:**
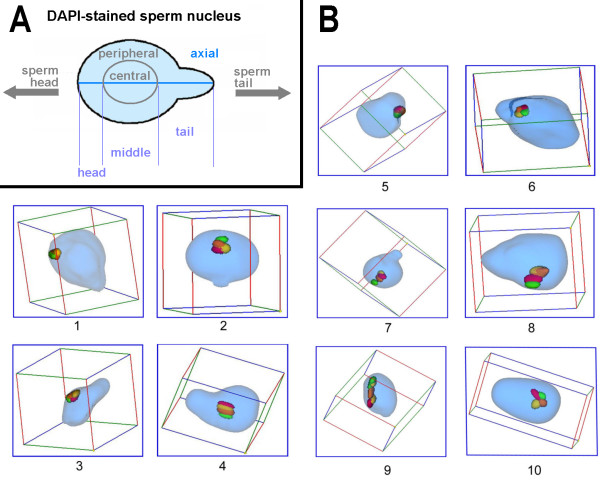
**a) Scheme of a human sperm nucleus after DAPI-staining and how it presented in this study.** The nucleus has a convexity where the sperm tail was attached at the cell. This convexity is shown here in exaggeration to make clear how the axial orientation of a nucleus was determined. As described in the text the sperm was divided into a central and a peripheral part, and deduced from that a head, middle and tail part could be defined. b) Examples for chromosomal positions in sperm: 1. chromosome 21 located in periphery and head of the sperm 2. chromosome 8 located in the center and head of the sperm. 3. X-chromosome located in periphery and middle of the sperm 4. chromosome 8 located in center and head of the sperm. 5. X-chromosome located in periphery and tail of the sperm. 6. X-chromosome located in center and tail of the sperm 7. chromosome 8 orientated axial 8. chromosome 10 orientated non-axial 9. chromosome 3 orientated linear 10. chromosome 11 orientated non-linear.

Here we present the first genome-wide MCB-based study on 3D-preserved interphase nuclei derived from sperm (Fig. 1). Previously, comparable FISH-studies on sperm were performed on flattened nuclei with the known disadvantages of possible artifacts due to transformation of a spherical into a pancake-like object [11,24-26] or even on decondensed nuclei with DNA looping out [27,28]. As for probes, the ones used were: whole, or arm-specific chromosome paintings, or centromeric probes [23-29]. We are aware of only one previous 3D-study on human sperm done by confocal microscope [22].

### Position, orientation and configuration of individual chromosomes

**Figure 2:**
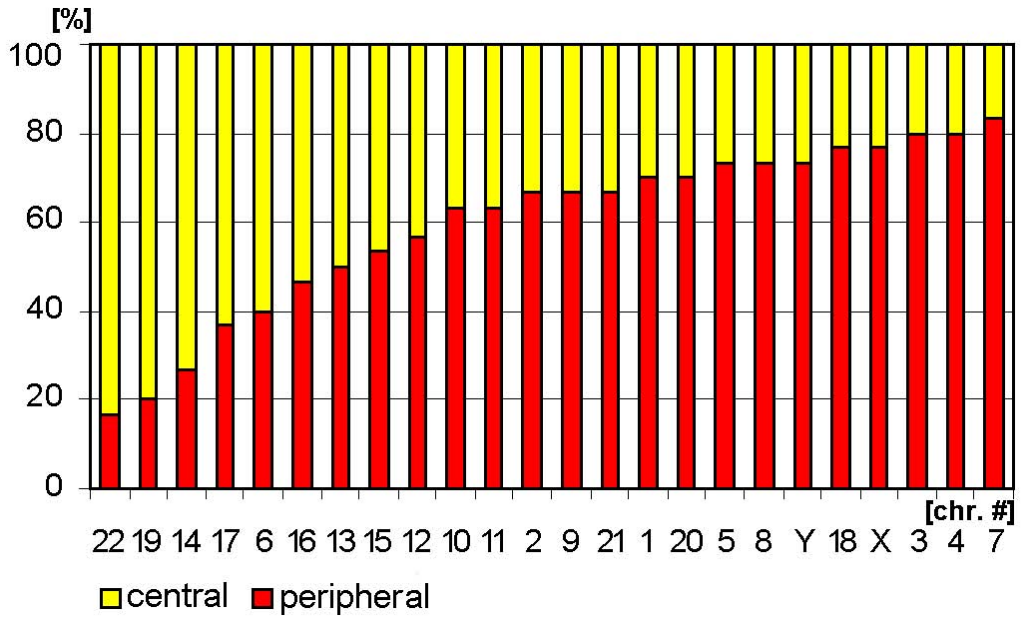
**Chromosomal distribution within the sperm cell evaluated according to the parameters 'central' (yellow) and 'peripheral' (red) localization (Fig. **1a**).** The chromosomes (= chr.#) are arranged from left to right corresponding to the probability of their whereabouts in a more peripheral or a more central way.

**Figure 3:**
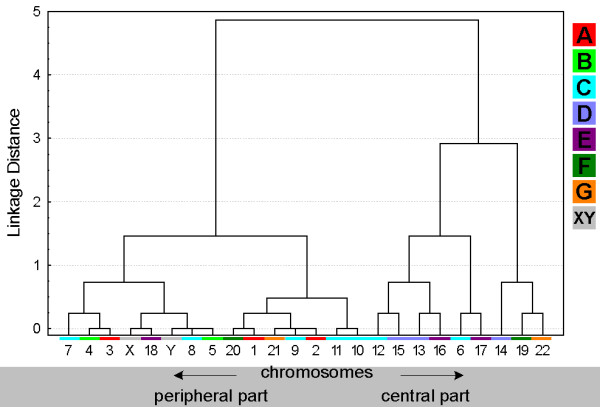
**Tree clustering algorithm of chromosomes localization on 'peripheral' and 'central' parts of the sperm deduced from the data of Fig. **2. The chromosome numbers in the X-axis are color-coded as shown in the legend on the right side of the diagram: A-group chromosomes in red, B-group in light-green, C-group in blue, D-group in violet, E-group in purple, F-group in dark-green, G-group in orange and gonosomes X and Y in gray.

**Figure 4:**
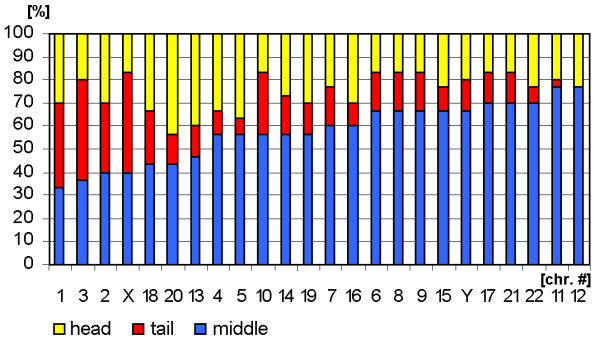
Distribution of the sperm chromosomes according to the parameters 'head' (yellow), 'tail' (red) and 'middle' (blue).

**Figure 5:**
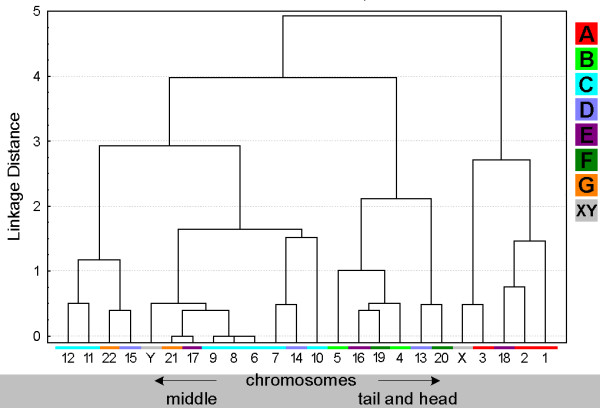
**Tree clustering algorithm of chromosomal localization according to 'middle' and 'tail and head'.** The figure is composed as described in the legend for Fig. 3.

**Figure 6:**
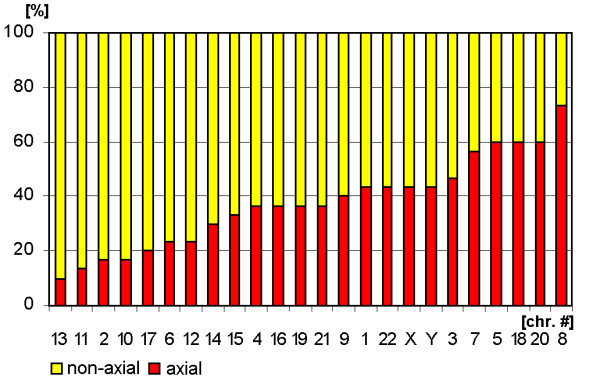
Arrangement of sperm chromosomes according to the parameters 'non-axial' (yellow) and 'axial' (red).

**Figure 7:**
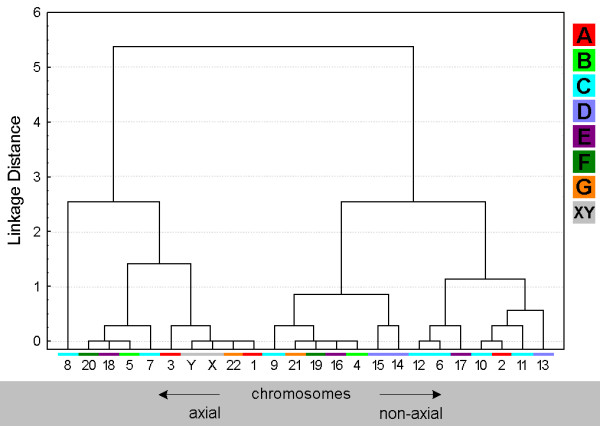
**Chromosomal arrangement according to 'axial' and 'non-axial' in a clustering algorithm.** The figure is composed as described in the legend for Fig. 3.

**Figure 8:**
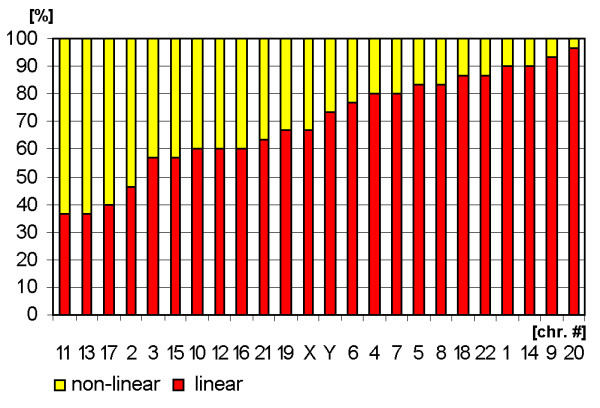
Sperm chromosomes arranged according to the parameters 'non-linear' (yellow) and 'linear' (red).

**Figure 9:**
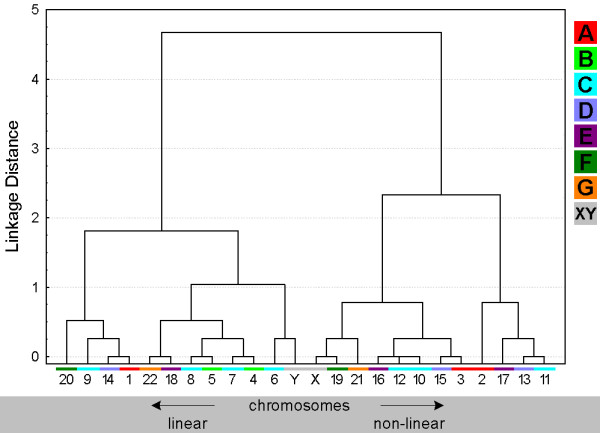
**Chromosomal arrangement according to 'linear' and 'non-linear' in a clustering algorithm.** The figure is composed as described in the legend for Fig. 3.

As summarized in Figures 2, 3, 4, 5, 6, 7, 8, 9 and Table 1 the statistical analysis revealed correlations between the investigated parameters central/peripheral, head/middle/tail, axial/non-axial, linear/non-linear and orientation of the chromosomal arms towards the sperm head, when analyzing the chromosomes by groups (see below).

**Table 1:** Orientation of chromosomes in sperm

Chromosomal orientation				
Chr.	Axial	p-arm	q-arm	non-axial

1	13 (43,3 ± 9,2)%	7	6	17 (56,7 ± 9,2)%
2	5 (16,7 ± 6,9)%	3	2	25 (83,3 ± 6,9)%
3	14 (46,7 ± 9,3)%	5	9	16 (53,3 ± 9,3)%
4	11 (36,7 ± 8,9)%	8	3	19 (63,3 ± 8,9)%
5	18 (60,0 ± 9,1)%	5	13	12 (40,0 ± 9,1)%
6	7 (23,3 ± 7,8)%	5	2	23 (76,7 ± 7,8)%
7	17 (56,7 ± 9,2)%	9	8	13 (43,3 ± 9,2)%
8	22 (73,3 ± 8,2)%	5	2	8 (26,7 ± 8,2)%
9	12 (40,0 ± 9,1)%	6	4	18 (60,0 ± 9,1)%
10	5 (16,7 ± 6,9)%	2	3	25 (83,3 ± 6,9)%
11	4 (13,3 ± 6,3)%	1	3	26 (86,7 ± 6,3)%
12	7 (23,3 ± 7,8)%	2	5	23 (76,7 ± 7,8)%
13	3 (10,0 ± 5,6)%	3	0	27 (90,0 ± 5,6)%
14	9 (30,0 ± 8,5)%	3	3	21 (70,0 ± 8,5)%
15	10 (33,3 ± 8,7)%	3	7	20 (66,7 ± 8,7)%
16	11 (36,7 ± 8,9)%	7	4	19 (63,3 ± 8,9)%
17	6 (20,0 ± 7,4)%	3	3	24 (80,0 ± 7,4)%
18	18 (60,0 ± 9,1)%	9	8	12 (40,0 ± 9,1)%
19	11 (36,7 ± 8,9)%	6	5	19 (63,3 ± 8,9)%
20	18 (60,0 ± 9,1)%	13	5	12 (40,0 ± 9,1)%
21	11 (36,7 ± 8,9)%	6	5	19 (63,3 ± 8,9)%
22	13 (43,3 ± 9,2)%	10	3	17 (56,7 ± 9,2)%
X	13 (43,3 ± 9,2)%	6	7	17 (56,7 ± 9,2)%
Y	13 (43,3 ± 9,2)%	5	8	17 (56,7 ± 9,2)%

Results are listed chromosome by chromosome of 30 sperm interphase nuclei, each. The chromosomal orientation is given for axial orientated chromosomes towards the head of the chromosome. Example: if the short arm was directed to the head of the sperm this nucleus was registered in the column 'p-arm'. Abbreviations: Chr. = chromosome; m = standard error; M = mean.

### Position of chromosomal sub-groups A-G

The ISCN provides the subdivision of human chromosomes in sub-groups A through G [30]. This is based on the chromosomal shape, size and centromeric position. In Figs. 3, 5, 7 and 9 clusters were formed from the obtained data and correlated with these chromosomal sub-groups.

***A- and B-group ***chromosomes are primarily located in the periphery (Fig. 3). While A-group is equally orientated towards the tail and head of the sperm, B-group is more frequently found in the middle and head part of the sperm (Fig. 5 and data not shown). These results are in concordance with previous studies for #1 [25,29], #2 [25] and #5 [23]. According to [29] the chromosome 2 is located more towards the tail of the sperm, which we could confirm by MCB.

Chromosomes of the ***C-group ***are positioned basically in the middle of sperm (Fig. 5). As visible in Fig. 3 C-group is localized preferentially in the periphery. Exceptions are here according to Fig. 3 chromosomes #6 and #12. It has to be noticed that for chromosome 6 already different data was reported [29], i.e. that chromosome 6 was observed to behave like all other chromosomes of the C-group as detected here. This discrepancy might be due to the fact that Zalenkaya and Zalensky (2004) [29] did their study on swollen and flattened slides and in parts with centromeric probes. On the other hand they report similar data as we found for chromosome 7 [29]. So further studies are necessary for a better understanding of the nuclear position of chromosome 6.

The ***D-group ***chromosomes can all be found in the central part of the sperm nucleus (Fig. 3). Also all three of them belong to the cluster which whereabouts are in the middle of the sperm (Fig. 5). Nonetheless, it is obvious that a tendency towards head or tail localization increases starting from #15, over #14 to #13. Hazzouri and coworkers found comparable data for chromosome 13, previously [22].

The ***E-group ***chromosomes #16 and #17 are localized in between middle and head and more towards the center of the sperm, as previously found by [29]. Distribution of chromosome #18 essentially differs from other chromosomes of this group as this chromosome is of preference to find on periphery in tail or head of the sperm cell (Figs. 3 and 5), as previously reported by others [26].

The ***F-group ***chromosomes #19 and #20 can both mainly be found towards the sperm head (Fig. 5 and data not shown). However, for the distribution towards periphery and center chromosomes #19 (central position) and #20 (peripheral position) differ clearly from each other. A similar observation was the outcome of this study for the G-group chromosomes 21 (peripheral position) and 22 (central position). On the other hand, both G-group chromosomes are located in the middle of the sperm (Figs. 3 and 5).

Both ***gonosomes ***show a similar distribution, i.e. they are found in the periphery of the sperm. The X-chromosome is localized towards the middle and tail of the sperm (Fig. 5 and data not shown), while the Y-chromosome can be found in the middle part.

### Orientation and configuration of chromosomal sub-groups A-G

All data concerning chromosomal orientation and configuration in sperm are summarized in Figs. 7 and 9 and Tab. 1.

Of the ***A-group ***chromosomes #1 and #3 tend to be localized more axial than #2. Surprisingly, the longest human chromosome #1 tends to be configured more linear rather than #2 and #3. Also the ***B-group ***chromosomes have a more linear configuration. #5 tends to be axial orientated, #4 non-axial.

The ***C-group ***is arranged in a non-axial way, in general. Chromosomes #7 and #8 are exceptions here. Chromosomes #6 to #9 tend to be more linear than the others of the C-group. ***D-group ***chromosomes turned out to be located non-axial. Chromosomes #13 and #15 are arranged non-linear, #14 more linear.

The ***E-group ***falls again in two different clusters: chromosomes #16 and #17 are configured non-axial and non-linear, while chromosome #18 behaves the other way round. The same holds true for the ***F-group***: #19 is non-axial and non-linear, #20 axial and linear arranged. In ***G-group ***similar things is to substitute by the same, even though the difference in axial/non-axial is not that expressed. Chromosome #22's configuration is more linear that of #21.

The ***gonosomes ***again behave very similar in terms of a more linear and neither expressed axial or non-axial behaviour.

A prevalence in orientation of p- or q-arm towards the sperm head could not be observed for half of the chromosomes (Tab. 1). Possible tendencies were observed for chromosomes #4, #6, #8, #13, #16, #20, #22 (p-arm) and #3, #5, #11, #12 and #15 (q-arm). However, as these results were obtained on 3 to 22 nuclei with axial orientated cells, they have to be considered as preliminary.

### Possible correlations with chromosome size

**Figure 10:**
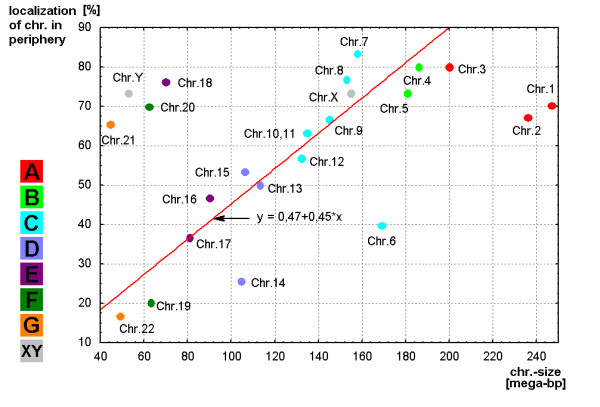
**Result of regression analysis for the correlation of chromosomal distribution [center/periphery (Y-axis)] and the chromosome (chr.) size in mega-base pair (mega-bp).** The regression is shown as a red line. The chromosomes are marked as dots in the corresponding colors of the groups A to G and gonosomes (X, Y), as specified in Figure 3.

As summarized in Fig. 10 a direct correlation of position of the chromosomes (from center to periphery) with their size can be found for most of the chromosomes. Apart from 8 chromosomes (#1, #2, #6. #14, #18, #20, #21 and Y) all other closely adjoin to the straight line of regression according with their size. The correlation level is high (correlation coefficient = 0.952).

### Possible correlations with gene density

**Figure 11:**
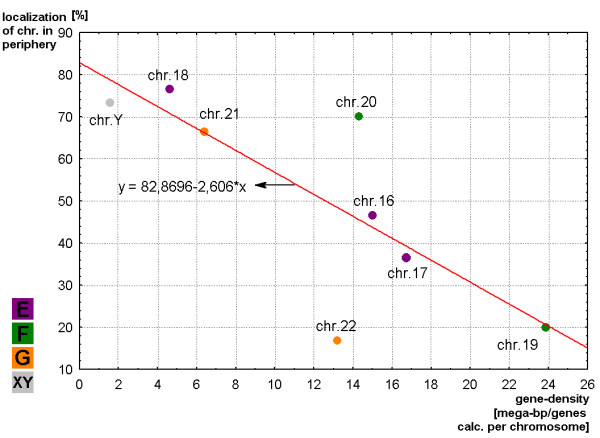
**Regression analysis for a similar correlation of small chromosomes F- to G-groups and Y chromosome as in Fig. **10. Here the chromosomal distribution [center/periphery (Y-axis)] are correlated with the gene density of each chromosome.

When arranging the smaller sized chromosomes (groups E, F, G and Y chromosome) then the gene density seems to have a more significant influence on the positioning of chromosomes in sperm. No such observation was possible for the larger chromosomes (results not shown). In Fig. 11 the smaller sized chromosomes are closely adjoined to the straight line of regression but comparing with the size dependence distribution relation has the opposite value. Chromosomes with a higher gene density are located in the center. Therefore, the Correlation Coefficient has negative value and equal to -0.983. The correlation level is high, however, chromosomes #20 and #22 do not completely fit.

## Conclusion

It could be demonstrated that MCB combined with S-FISH is a powerful tool for a three-dimensional analysis of chromosome position in sperm interphase nuclei. The topology in interphase nucleus of human is organized in a non-random way driven by chromosome size and gene density. This is now not only clear for lymphocytes but also for sperm. Interestingly for most of the chromosomes the distribution of the territories seem to be similar in sperm and lymphocytes [31] apart from the acrocentric chromosomes as previously discussed [11].

Further combined application of multicolor banding with three-dimensional analysis in various tissues will provide to a better understanding of interphase architecture in human. Future studies in sperm of patients with unexplained fertility problems may characterize yet unknown mechanisms of infertility, as Cremer and colleagues postulated in 2004 [32]: the nuclear architecture may be an integrated part of the epigenetic mechanisms.

## Methods

### Human sperm

Human sperm sample was collected in a sterile container after 3 days of sexual abstinence from a fertile, 30 year-old man with normal seminal parameters and normal karyotype. After liquefaction at room temperature, the samples was washed three times in 1 × phosphate-buffered saline by centrifugation (5 min at 2000 rpm) and fixed in fresh fixative (1:3 glacial acetic acid: methanol) [33].

### Suspension-fluorescence in situ hybridization (S-FISH)

S-FISH on interphase sperm cells was done as previously reported [11]. 30 cells were evaluated per chromosome i.e. overall 720 interphase nuclei were analyzed. The same number of evaluated cells per chromosome was chosen in comparable previous studies [11,29].

### Evaluation

Analysis of chromosomal position in sperm included several parameters. The localization of chromosomes in periphery or center was determined. For that the sperm was divided into two spheres and 50% of the radius of cell was defined as central, the remainder as peripheral (Figs. 1, 2, 3). As the sperm axis can be determined in DAPI-staining (see [11] and Fig. 1a) it is also possible to define chromosomal localization as head, middle and tail parts of the sperm (Figs. 1; 4, 5). Moreover, it was determined if the chromosomes located along the longitudinal axis of the sperm or not. Chromosomes were defined as axial when the deviation was less the 45° and/or the chromosome was not entangled (Figs. 1; 6, 7). The latter was also registered by distinguishing linear and non-linear configuration of chromosomes (Figs. 1; 8, 9). Finally, the orientation concerning the short p- and long q-arms of sperm chromosomes towards the head of sperm was recorded (Fig. 1; Tab. 1).

### Statistics

Statistical analysis was performed using Student's t-test and One Way ANOVA (Analysis of Variance) to determine significant differences of chromosome's arrangement in sperm. Statistical significance was defined as p < 0.05.

Estimation of similarity in a position and orientations between various chromosomes was done with the application of cluster analysis (Figs. 3, 5, 7 and 9). The purpose of this algorithm is to correlate chromosomes within clusters, which are depicted as hierarchical trees. Linkage distances between chromosomes are computed based of Euclidean distances. This is simply the geometric distance in the multidimensional space. For analysis of dependence of chromosomes distribution on periphery and the center of sperm from the length and relative gene density of chromosomes, the regression analysis was used (Figs. 10, 11).

## Competing interests

The authors declare that they have no competing interests.

## Authors' contributions

MM, FH and SB did 3-D-FISH in human sperm. FP and SB provided and prepared the human sperm pellet. FH, KM and AW adapted the S-FISH protocol for MCB-probes. FP, AW, RA, IS, TL have been involved in drafting the manuscript and revising it critically for important intellectual content.
